# Acute Administration of Metformin Protects Against Neuronal Apoptosis Induced by Cerebral Ischemia-Reperfusion Injury *via* Regulation of the AMPK/CREB/BDNF Pathway

**DOI:** 10.3389/fphar.2022.832611

**Published:** 2022-04-01

**Authors:** Ke Liu, Lulu Li, Zhijun Liu, Gang Li, Yanqing Wu, Xingjun Jiang, Mengdie Wang, Yanmin Chang, Tingting Jiang, Jianheng Luo, Jiahui Zhu, Hongge Li, Yong Wang

**Affiliations:** ^1^ Department of Neurology, Union Hospital, Tongji Medical College, Huazhong University of Science and Technology, Wuhan, China; ^2^ Department of Neurology, People’s Hospital of Zhengzhou, People’s Hospital of Henan University of Chinese Medicine, Zhengzhou, China

**Keywords:** cerebral ischemia-reperfusion injury, metformin, AMP-activated protein kinase, neuronal apoptosis, brain-derived neurotrophic factor

## Abstract

Metformin is a first-line anti-diabetic agent with a powerful hypoglycemic effect. Several studies have reported that metformin can improve the prognosis of stroke patients and that this effect is independent of its hypoglycemic effect; however, the specific mechanism remains unclear. In this research, we explored the effect and specific mechanism of metformin in cerebral ischemia-reperfusion (I/R) injury by constructing a transient middle cerebral artery occlusion model *in vivo* and a glucose and oxygen deprivation/reoxygenation (OGD/R) model *in vitro*. The results of the *in vivo* experiments showed that acute treatment with low-dose metformin (10 mg/kg) ameliorated cerebral edema, reduced the cerebral infarction volume, improved the neurological deficit score, and ameliorated neuronal apoptosis in the ischemic penumbra. Moreover, metformin up-regulated the brain-derived neurotrophic factor (BDNF) expression and increased phosphorylation levels of AMP-activated protein kinase (AMPK) and cAMP-response element binding protein (CREB) in the ischemia penumbra. Nevertheless, the above-mentioned effects of metformin were reversed by Compound C. The results of the *in vitro* experiments showed that low metformin concentrations (20 μM) could reduce apoptosis of human umbilical vein endothelial cells (HUVECs) under OGD/R conditions and promote cell proliferation. Moreover, metformin could further promote BDNF expression and release in HUVECs under OGD/R conditions *via* the AMPK/CREB pathway. The Transwell chamber assay showed that HUVECs treated with metformin could reduce apoptosis of SH-SY5Y cells under OGD/R conditions and this effect could be partially reversed by transfection of BDNF siRNA in HUVECs. In summary, our results suggest that metformin upregulates the level of BDNF in the cerebral ischemic penumbra *via* the AMPK/CREB pathway, thereby playing a protective effect in cerebral I/R injury.

## Introduction

Acute ischemic stroke is the most prevailing type of cerebrovascular disease. Due to its high morbidity, mortality, and disability, it has received widespread attention and imposes a substantial burden on national health and the social economy. Intravenous recombinant tissue plasminogen activator thrombolysis (National Institute of Neurological Disorders and Stroke rt-PA Stroke Study Group, 1995) and mechanical arterial thrombectomy (Goyal et al., 2015; Campbell et al., 2015; Berkhemer et al., 2015) are currently the most efficient treatments for opening occluded blood vessels and restoring cerebral blood perfusion. However, rapid reperfusion after cerebral ischemia can cause secondary damage, named cerebral ischemia-reperfusion (I/R) injury, which is even more serious than simple cerebral ischemic injury. Cerebral I/R injury often results in the death of a large number of neurons, leading to widespread cerebral infarction and severe cognitive dysfunction (Lin et al., 2016). Therefore, there is an urgent need to identify the pathogenesis of cerebral I/R injury and find effective therapies.

The neurovascular unit is a concept that emphasizes the interaction between the cerebrovascular system and brain tissue cells, which contains neurons, vascular endothelial cells (ECs), glial cells, and the basement membrane (Iadecola, 2017). As one of the central components of the neurovascular unit, ECs can not only provide blood flow and nutrients for the brain but also play a neuroprotective role by secreting neurotrophic factors. This effect of ECs is known as nerve-vascular coupling (Guo et al., 2008; Alhusban et al., 2013; Fouda et al., 2017). Neurotrophic factors play a crucial role in the nervous system *via* regulating the survival and differentiation of neurons (Mizui et al., 2016). Brain-derived neurotrophic factor (BDNF), as a member of neurotrophic factors, has powerful neurogenesis, neuroprotection, and angiogenesis effects (Caporali and Emanueli, 2009; Greenberg et al., 2009) and is also relevant to learning and memory (Tyler et al., 2002; Bekinschtein et al., 2014). Some studies have found that BDNF is involved in the nerve-vascular coupling between ECs and nerve cells (Guo et al., 2008). Furthermore, several studies have shown that BDNF can be upregulated and has an important neuroprotective effect after experimental stroke (Guo et al., 2008; Alhusban et al., 2013; Fouda et al., 2017).

Metformin, a first-line biguanide drug, is widely used in patients with type 2 diabetes because of its hypoglycemic effect. Several clinical studies have revealed that metformin can also improve the prognosis of stroke patients (Mima et al., 2016) and decrease the risk of long-term cardiovascular events (UK Prospective Diabetes Study (UKPDS) Group, 1998; Nathan, 1998; Selvin and Hirsch, 2008). Furthermore, the activation of AMP-activated protein kinase (AMPK) plays a dominant role in the protective effect of metformin (Alexander et al., 2013). Related studies have shown that AMPK is a vital endogenous defense factor that plays a protective effect in ischemic stroke *via* alleviating neuroinflammation, reducing oxidative stress, improving mitochondrial dysfunction, and inhibiting cell apoptosis (Jiang et al., 2018). Metformin has also been confirmed in many studies to promote phosphorylation of AMPK protein, thereby exerting neuroprotective effects by activating downstream molecules that reduce the adverse influences of cerebral I/R injury (Ashabi et al., 2014; Venna et al., 2014; Ashabi et al., 2015).

The specific mechanism by which metformin alleviates cerebral I/R injury remains ambiguous. Nevertheless, various *in vivo* and *ex vivo* experimental models have shown that metformin can promote BDNF expression (Patil et al., 2014; Han et al., 2018; Keshavarzi et al., 2019). Subsequently, we hypothesized that metformin can upregulate BDNF expression to exert a neuroprotective effect, thus alleviating cerebral I/R injury in an experimental model of ischemic stroke. Our study aimed to further understand the mechanism by which metformin improves cerebral I/R injury and to provide new evidence and support for metformin as a therapy that can be synchronized with vascular recanalization as a treatment for acute cerebral infarction.

## Materials and Methods

### Animals

Male specific pathogen-free Sprague-Dawley rats (age, 6–8 weeks; weight, 280–300 g) were purchased from Sipeifu Biotechnology Co., Ltd. (Beijing, China). During the experiments, two rats per cage were housed in a specific pathogen-free environment (temperature 21–24°C, humidity 55%–65% and a 12-h light/dark cycle) with free access to food and water. All experiments were performed in accordance with the Experimental Animal Management Committee of Tongji Medical College of Huazhong University of Science and Technology (IACUC Number: 2499).

### Construction of a tMCAO Model and Drug Administration *in vivo*

**FIGURE 1:**
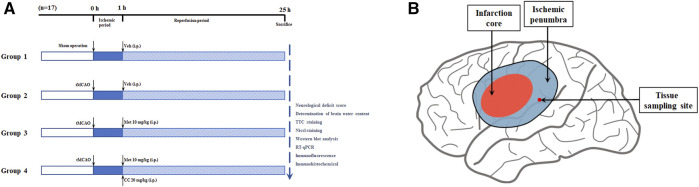
Schematic diagram of animal experiment groupings and ischemic penumbra. **(A)** The schematic diagram of animal experiment groupings (*n* = 17 in each group). Abbreviations: tMCAO, transient middle cerebral artery occlusion; Veh, vehicle; Met, metformin; CC, Compound C. **(B)** The schematic diagram of ischemic penumbra.

After anesthesia with 2% sodium pentobarbital (40 mg/kg, i.p.), a transient middle cerebral artery occlusion (tMCAO) rodent model was constructed based on previous studies (Yuan et al., 2016). In brief, a neck incision was made, the common carotid artery was exposed, and the internal carotid and external carotid arteries were then carefully separated. Next, the blood flow in the right middle cerebral artery was blocked with a 4-0 monofilament nylon suture (Cinontech, Beijing, China) coated with poly-L-lysine. Thereafter, the sutures were pulled out after 60 min of ischemia. At the beginning of the reperfusion period, rats in each group were administered metformin (10 mg/kg, i.p.; Sigma-Aldrich, United States) and Compound C (CC, 20 mg/kg, i.p.; an AMPK inhibitor, APExBIO, United States), according to the experimental design (the specific experimental groups are shown in Figure 1). The concentration and mode of administration of metformin and Compound C were based on previous research by Jiang et al. (2014). The sham operation group underwent the same surgical procedure except for the insertion of the suture and received an equal volume of the vehicle at the beginning of the reperfusion period. All rats were euthanized 24 h after the reperfusion period, then the brain tissues of the ischemic core, the ischemic penumbra, and the contralateral cerebral hemisphere were collected and stored at -80°C for later use. The body temperature of rats was maintained at 37.1 ± 0.5°C from after anesthesia to before euthanasia with a heating pad.

### Neurological Deficit Score

After successful modeling, all rats were evaluated for neurological deficits using the Zea-Longa five-point scoring system (Longa et al., 1989) before they were euthanized: 0 points, normal; 1 point, weakness of the left forelimb and incomplete extension; 2 points, turning to the left side when walking; 3 points, cannot bear weight on the left side; and 4 points, no spontaneous activity, and disturbed consciousness.

### TTC Staining

After the rats were euthanized, the volume of cerebral infarction was measured using the 2,3,5-triphenyltetrazolium chloride (TTC) staining method. Briefly, the brain was removed and rapidly frozen in a refrigerator at -20°C for 20 min and cut into 6–7 slices of 2-mm thickness. Next, the slices were stained with 2% TTC solution for 20 min. The volume of cerebral infarction was measured *via* an Image-Pro Plus analysis system.

### Determination of Brain Water Content

We used our previous experiments (Wang et al., 2018) to determine the water content of the brain tissue and evaluate brain edema in the rats in each group. After the brains were taken out and the cerebellum and brain stem were removed, wet weights (WW) were obtained. Next, the dry weights (DW) were obtained after the brain was dried in an oven at 100°C for 24 h. The brain water content was calculated *via* the following formula: brain water content (%) = (WW-DW)/WW×100%.

### Immunohistochemical Analysis

After successful modeling, several rats were selected for anesthesia, followed by perfusion with normal saline and 4% paraformaldehyde. The brain was then taken out, embedded in paraffin, and cut coronally into 5-μm thick brain slices. Next, paraffin sections of the brain tissues in each group were deparaffinized by xylene and then rehydrated with gradient alcohol (100%, 95%, 80%, and 75%). The tissue sections were sequentially subjected to antigen retrieval (citric acid antigen retrieval buffer, high temperature, and pressure conditions), blocking endogenous peroxidase (3% H_2_O_2_ solution, protected from light for 25 min), and serum sealing (5% BSA solution, 40 min). The brain slices were then incubated with the prepared BDNF primary antibody (1:500; Abcam, United States) at 4°C for 18–24 h. After washing, brain slices were incubated with the secondary antibody of the corresponding species for 2 h. After washing again, the brain slices were developed by 3,3′-diaminobenzidine and counterstained with hematoxylin. Finally, after dehydration with gradient alcohol and transparent xylene, the brain slices were mounted with neutral gum for microscopic examination. Images were obtained using an Olympus photomicroscope (Nikon, Tokyo, Japan).

### Nissl Staining

Paraffin sections were deparaffinized and rehydrated as previously described. Next, the brain slices were stained with Nissl solution, washed with distilled water, dried, and mounted with neutral gum. Finally, the slices were observed and images were acquired using the Olympus photomicroscope.

### Cell Culture

Human umbilical vein endothelial cells (HUVECs) and SH-SY5Y cells were purchased from the China Center for Type Culture Collection (Hubei, China). Both types of cells were cultured in Dulbecco’s modified Eagle’s medium (DMEM; Gibco, Waltham, MA, United States) containing 10% fetal bovine serum and 1% penicillin-streptomycin solution (Solarbio, Beijing, China) and incubated in a humidified incubator filled with 5% CO_2_ and 95% O_2_ at 37°C.

### Transfection With Small Interfering RNAs

BDNF small interfering RNA (siRNA) and negative control siRNA were obtained from Genomeditech (Shanghai, China). The BDNF siRNA sequence was as follows: 5′-GAA​UUG​GCU​GGC​GAU​UCA​UAA-3′ and 3′-CUU​AAC​CGA​CCG​CUA​AGU​AUU-5’ (the blocking effect of BDNF siRNA is shown in Supplementary Figure S3). The HUVECs were cultured in the upper chamber. After the cells grew normally and the confluence reached 30–50%, transfection was performed according to the manufacturer’s protocols (RiboBio, Guangzhou, China) with a final concentration of 50 nM siRNA. The cells were used for the subsequent Transwell chamber assay 48 h after the transfection.

### Construction of an OGD/R Model and Drug Administration *in vitro*

For oxygen-glucose deprivation (OGD), the cells were discarded from the original medium and rinsed twice with phosphate-buffered saline, glucose-free DMEM was added and then cultured in a humidified anaerobic incubator (containing 1% O_2_, 5% CO_2_, and 94% N_2_) at 37°C to simulate the ischemic environment *in vitro*. In this study, HUVECs and SH-SY5Y cells were maintained under OGD conditions for 2, 4, 6, or 8 h and 0.5, 1, 2, or 4 h, respectively. The culture medium was then replaced with complete DMEM and the cells were placed in a normal incubator (containing 95% O_2_ and 5% CO_2_) at 37°C for reoxygenation for 12 h to mimic reperfusion *in vitro*. At the beginning of the reoxygenation phase, metformin (0, 5, 10, 20, 50, and 100 μM), AICAR (an AMPK agonist, 500 μM; MedChemExpress, China), Compound C (10 μM), and KG-501 (a CREB inhibitor, 25 μM; Sigma-Aldrich, United States) were added to the culture medium to treat HUVECs, and BDNF (0, 10, 20, 50, and 100 ng/ml; R&D Systems, United States) was added to treat SH-SY5Y cells in different groups. All cells were collected for later experiments at the end of the reoxygenation period.

### Cell Counting Kit-8 Assay

Approximately 5,000 cells/well were seeded in a 96-well plate. After the cell processing of each well was completed, the original medium was discarded, and 100 μL of new DMEM and 10 μL of Cell Counting Kit-8 (CCK-8) solution (Dojindo Technologies, Japan) were added and incubated for 2 h. Finally, the absorbance of each well was measured at 450 nm by a microplate reader for subsequent analysis.

### Western Blot Analysis

The brain tissue samples in the ischemic penumbra, and the processed HUVECs were homogenized in RIPA lysis buffer and then centrifuged to obtain tissue and cell proteins. The protein concentration was measured using the BCA protein assay kit (Beyotime, China), and 20 μg of protein was separated for electrophoresis. Proteins were then transferred to a 0.45-μm PVDF membrane (Merck, Germany). After blocking with 5% non-fat milk for 1 h at 18–20°C, the membranes were incubated with the corresponding primary antibodies at 4°C for 18–24 h. After washing three times, the membranes were incubated with secondary antibodies of the corresponding species for 1 h. Finally, the protein bands were visualized by chemiluminescence and analyzed using ImageJ software. The primary antibodies used in this study were as follows: AMPK (1:1000; CST, United States), p-AMPK (1:1000; Abcam, United States), CREB (1:1000; CST), p-CREB (1:1000; Abcam), BDNF (1:1000; Abcam), and GAPDH (1:3000; Proteintech, China).

### Enzyme-Linked Immunosorbent Assay

Plasma samples were obtained by centrifuging the blood samples at 1,200 g for 10 min. To detect the BDNF concentration in the plasma of rats and culture supernatant of HUVECs, a BDNF enzyme-linked immunosorbent assay (ELISA) kit (Multi Sciences, China) was used according to the manufacturer’s protocols.

### Real-Time Quantitative PCR

TRIzol and reverse transcription kits (Takara, Japan) were used for RNA extraction and reverse transcription according to the manufacturer’s instructions. ChamQ SYBR qPCR Master Mix (Vazyme, China), cDNA, and primers were mixed in the polymerase chain reaction (PCR) plate, after which the transcription level of BDNF was analyzed using the StepOnePlus real-time PCR System. The primers used in this experiment were as follows: actin (human, 5′-3′, forward/reverse), AGA​GCT​ACG​AGC​TGC​CTG​AC and AGC​ACT​GTG​TTG​GCG​TAC​AG; BDNF (human, 5′-3′, forward/reverse) TGT​TGG​ATG​AGG​ACC​AGA​AAG​TT and GCC​TCC​TCT​TCT​CTT​TCT​GCT​GG; actin (rat, 5′-3′, forward/reverse) TTG​TCA​CCA​ACT​GGG​ACG​ATA​TGG and GGG​TGT​TGA​AGG​TCT​CAA​ACA​TG; BDNF (rat, 5′-3′, forward/reverse) CAGGGGCATAGACAAAAG and CTT​CCC​CTT​TTA​ATG​GTC.

### Immunofluorescence

Immunofluorescence was used to measure BDNF expression in ECs in the penumbra of cerebral ischemia. Paraffin sections of brain tissues were deparaffinized and rehydrated, and antigen retrieval and serum blocking were performed as described above. The cells were then incubated at 4°C with the CD31 (1:50; R&D Systems, United States) and BDNF (1:500; Abcam, United States) primary antibodies for 18–24 h. After washing, the samples were incubated with a fluorescent secondary antibody of the corresponding species for 1 h at 18–20°C. Finally, after DAPI staining, the slices were mounted with anti-fluorescence quenching mounting tablets and examined under a microscope.

Immunofluorescence staining was also performed to detect BDNF expression in HUVECs. The cells were seeded on slides and processed accordingly. After fixation, permeabilization and serum blocking, the cell slides were incubated at 4°C with the BDNF primary antibodies (1:500; Abcam, United States) for 18–24 h. After washing, the cell slides were incubated with a fluorescent secondary antibody of the corresponding species for 1 h at 18–20°C. Finally, after DAPI staining, the cell slices were mounted with anti-fluorescence quenching mounting tablets and examined under a microscope.

### Analysis of Apoptosis

Apoptosis of neurons in the ischemic penumbra area *in vivo* and apoptosis of HUVECs and SH-SY5Y cells under OGD/R conditions *in vitro* was detected using a one-step TdT-mediated dUTP nick-end labeling (TUNEL) apoptosis assay kit (Beyotime, China) according to the manufacturer’s instructions. Related steps are referred to in the immunofluorescence section. The primary antibody used was as follows: NeuN (1:100; Proteintech, China).

Apoptosis of HUVECs and SH-SY5Y cells under OGD/R conditions was also detected using the Annexin V-FITC/PI apoptosis kit (Multi Sciences, China). After the staining, the cells were immediately detected using a flow cytometer (BD Biosciences, United States). The results were analyzed by FlowJo software.

The Transwell chamber assay was used to explore the effect of metformin-treated HUVECs on apoptosis of SH-SY5Y cells under OGD/R conditions. Briefly, HUVECs were seeded in the upper chamber and exposed to OGD conditions for 5 h after transfection with siRNA. SH-SY5Y cells were then seeded in the lower chamber and co-cultured with HUVECs under OGD conditions for 1 h (from hour 5 to hour 6). Subsequently, HUVECs located in the upper chamber were treated accordingly at the beginning of the reoxygenation period (schematic representations of each treatment group are shown in Figure 8Aand schematic diagram for transwell chamber assay is shown in Supplementary Figure S2). After a 12-h reoxygenation period, the cells in the lower chamber were collected and apoptosis rate was detected by flow cytometry and TUNEL staining.

### Statistical Analysis

All data were analyzed using GraphPad Prism 8 software and expressed as the mean ± SD. Comparisons between two groups were performed by the independent-samples *t*-test. Pairwise comparisons between multiple groups were performed by ordinary one-way analysis of variance and Tukey’s multiple comparisons test. Neurological deficit scores were compared between groups using the Mann-Whitney *U* test. *p* < 0.05 was considered statistically significant.

## Results

### Metformin Alleviated Cerebral Edema, Reduced the Volume of Cerebral Infarction, Improved the Neurological Deficit Score, and Ameliorated Neuronal Apoptosis in the Ischemic Penumbra *in vivo*

**FIGURE 2:**
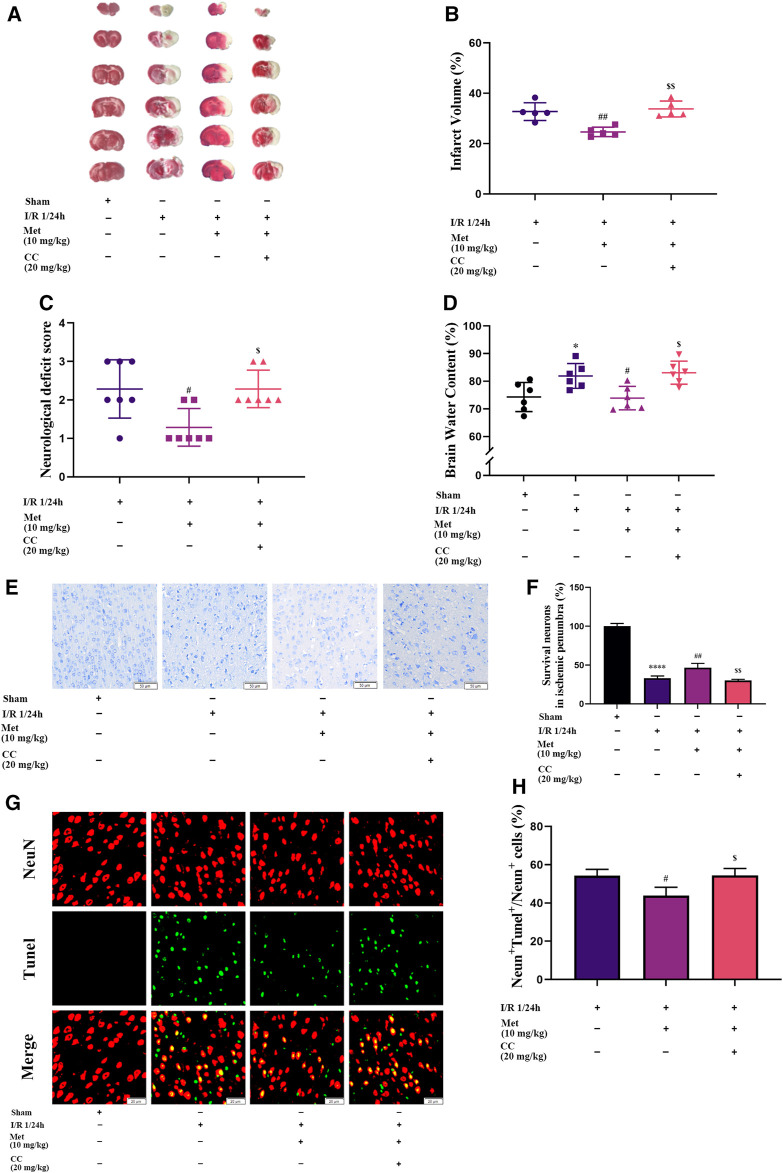
Metformin alleviates cerebral edema, reduces cerebral infarct volume, improves the neurological deficit score, and ameliorates neuronal apoptosis in the ischemic penumbra. However, Compound C significantly reduces the above-mentioned effects of metformin. **(A,B)** Cerebral infarct volume was assessed by TTC staining (*n* = 5). **(C)** The neurological deficit score was assessed by the Zea-Longa five-point scoring system (*n* = 7). **(D)** Brain edema was evaluated by measuring the brain water content (*n* = 6). **(E–H)** Survival and apoptosis of neurons around the ischemic penumbra were evaluated by Nissl staining (*n* = 3, bar = 50 μm) and TUNEL staining (*n* = 3, bar = 20 μm), respectively. (*p < 0.05, ****p < 0.0001 vs. sham; ^#^ *p* < 0.05, ^##^ *p* < 0.01 vs. I/R 1/24 h; ^$^ *p* < 0.05, ^$$^ *p* < 0.01, vs. I/R 1/24 h + Met).

To explore the function of metformin on cerebral I/R injury *in vivo*, brain edema, the volume of cerebral infarction, and the neurological deficit score were subjected to statistical analysis after the reperfusion period. As shown in Figure 2C, treatment with metformin dramatically improved the neurological deficit score after cerebral I/R injury *in vivo*. In addition, after the administration of metformin, cerebral edema improved remarkably (Figure 2D). TTC staining revealed that the volume of cerebral infarction was also dramatically reduced by treatment with metformin (Figures 2A,B). Nissl staining implied that the number of normal neurons in the ischemic penumbra was significantly higher in the metformin group than in the operation group (Figures 2E,F). Moreover, TUNEL staining showed that apoptosis of neurons in the metformin group was markedly reduced (Figures 2G,H). However, it is intriguing that treatment with Compound C at the same time as treatment with metformin reversed the aforementioned positive effects. This finding suggests that the protective role of metformin in cerebral I/R injury is at least partially mediated by activation of AMPK.

### Metformin Improved Cerebral I/R Injury *via* Regulating BDNF Expression *in vivo*

**FIGURE 3:**
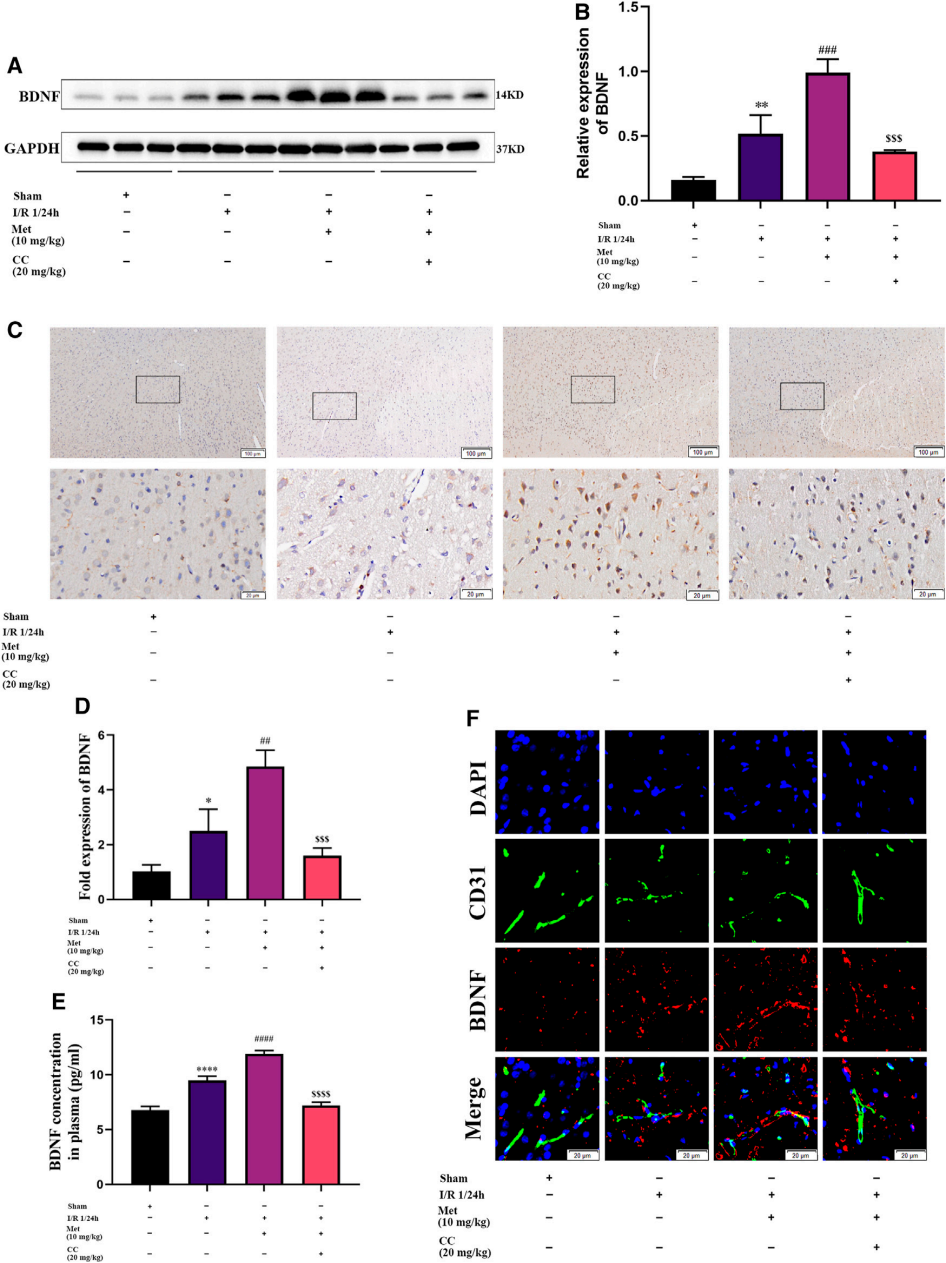
Metformin improves cerebral ischemia/reperfusion (I/R) injury by regulating the expression of BDNF. **(A–C)** BDNF protein expression in the ischemic penumbra area was evaluated by western blotting and immunohistochemistry (*n* = 3, bar = 100 μm/20 μm). **(D)** The BDNF transcription level in the ischemic penumbra area was assessed by RT-qPCR (*n* = 3). **(E)** The BDNF concentration in the plasma assessed by ELISA (*n* = 3). **(F)** BDNF expression in endothelial cells in the ischemic penumbra was evaluated by immunofluorescence staining (*n* = 3, bar = 20 μm). (*p < 0.05, **p < 0.01, ****p < 0.0001 vs. sham; ^##^ *p* < 0.01, ^###^ *p* < 0.001, ^####^ *p* < 0.0001 vs. I/R 1/24 h; ^$$$^ *p* < 0.001, ^$$$$^ *p* < 0.0001 vs. I/R 1/24 h + Met).

We speculated that metformin plays an active role in cerebral I/R injury *via* regulating BDNF expression. To verify our hypothesis, we used quantitative PCR and western blotting to measure the mRNA and protein levels of BDNF in the cortex of the cerebral ischemic penumbra, and ELISA was used to assess the BDNF concentration in the plasma of the rats in each group. Western blotting revealed that administration of metformin further increased BDNF expression (Figures 3A,B), and ELISA showed that metformin treatment also upregulated BDNF concentrations in the plasma (Figure 3E). The results of quantitative PCR showed that metformin also increased the level of BDNF transcription (Figure 3D), indicating that metformin regulated BDNF expression at the transcription level. Furthermore, consistent with the above results, brain slice immunohistochemistry indicated that administration of metformin significantly upregulated BDNF expression in the penumbra of cerebral ischemia (Figure 3C). Interestingly, immunofluorescence staining of brain slices revealed that metformin can act on ECs to promote BDNF expression in ECs. However, treatment with Compound C inhibited the metformin-mediated upregulation of BDNF expression (Figure 3F). Therefore, metformin plays a protective role in cerebral I/R injury at least in part *via* regulating BDNF expression in ECs at the transcriptional level, with AMPK activation as part of the process.

### AMPK and CREB Were Involved in Regulating BDNF Expression in Cerebral I/R Injury

**FIGURE 4:**
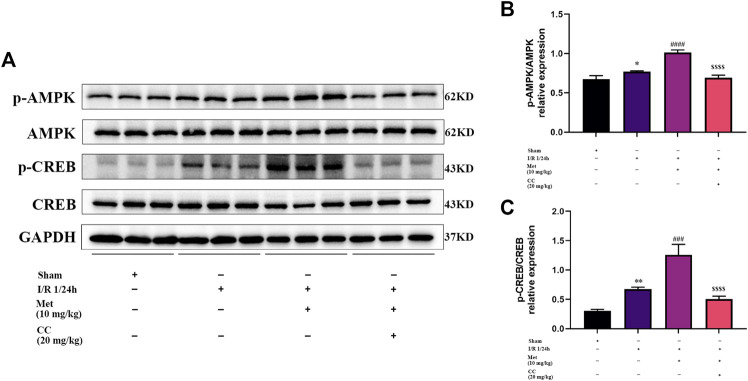
AMPK and CREB are involved in the regulation of BDNF expression by metformin in cerebral ischemia-reperfusion (I/R) injury. **(A–C)** The phosphorylation levels of AMPK and CREB proteins was detected by western blotting (*n* = 3). (^*^ *p* < 0.05, ^**^ *p* < 0.01 vs. sham; ^###^ *p* < 0.001, ^####^ *p* < 0.0001 vs. I/R 1/24 h; ^$$$$^ *p* < 0.0001 vs. I/R 1/24 h + Met).

To further explore whether AMPK was involved in regulating BDNF expression, we detected the AMPK and p-AMPK expression levels in the brain tissue around the ischemic penumbra by western blotting. The results showed that metformin significantly increased the phosphorylation of AMPK, and this effect was inhibited by Compound C (Figures 4A,B). Furthermore, previous studies have found that transcriptional induction of BDNF was closely related to the CREB protein family (Tao et al., 1998) and have suggested that metformin could induce BDNF expression at the transcriptional level. Consequently, we speculated that CREB might participate in the regulation of BDNF expression in cerebral I/R injury, and tested this possibility by western blotting to detect CREB and p-CREB protein levels in the ischemic penumbra. We found that metformin could increase the phosphorylation level of CREB, and this phenomenon could also be reversed by Compound C (Figures 4A,C). Therefore, it could be concluded that metformin regulated BDNF expression in cerebral I/R injury at least in part by regulating the phosphorylation of AMPK and CREB.

### Metformin Upregulated BDNF Expression and Release, Promoted Proliferation of HUVECs, and Inhibited Cell Apoptosis Under OGD/R Conditions *in vitro*

**FIGURE 5:**
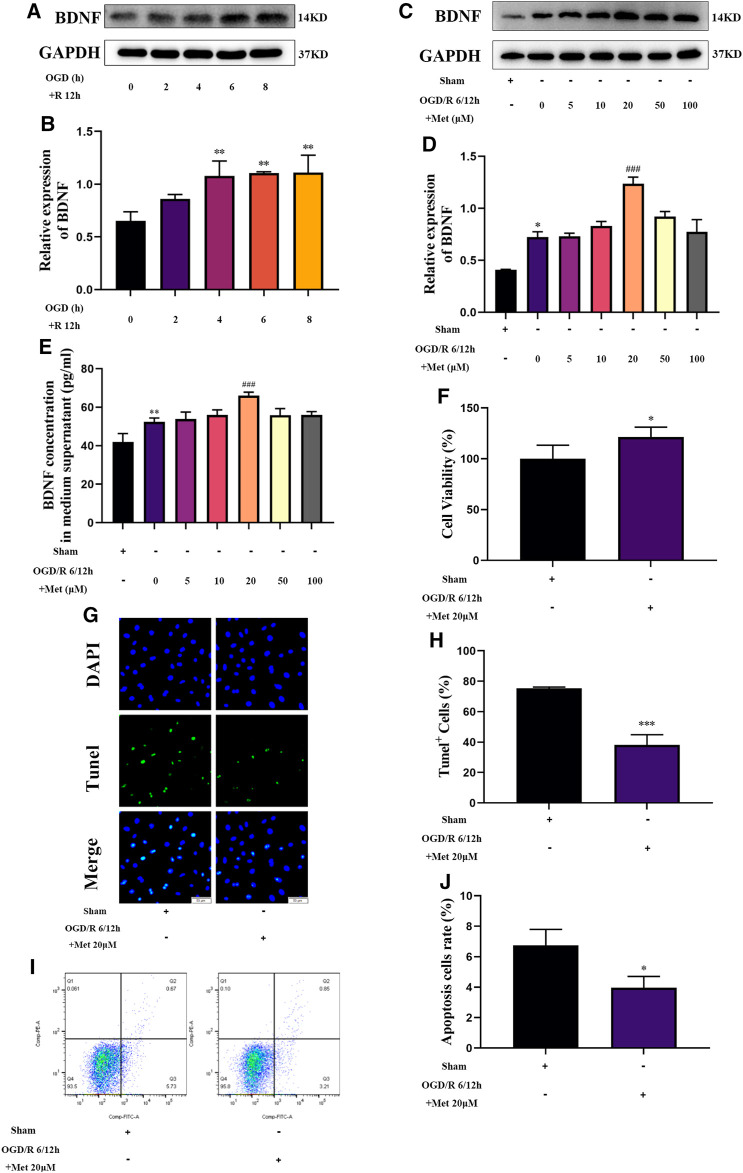
Metformin upregulates BDNF expression and release, promotes HUVECs proliferation, and inhibits cell apoptosis under OGD/R conditions. **(A–D)** The BDNF expression level of metformin-treated HUVECs was detected by western blotting (*n* = 3). **(E)** The BDNF concentration in the culture supernatant of metformin-treated HUVECs was detected by ELISA (*n* = 3). **(F)** Proliferation of HUVECs was detected by CCK-8 assay (*n* = 5). **(G–J)** Apoptosis of HUVECs was detected by TUNEL staining (*n* = 3, bar = 50 μm) and flow cytometry (*n* = 3). (^*^ *p* < 0.05, ^**^ *p* < 0.01 vs. sham; ^#^ *p* < 0.05, ^###^ *p* < 0.001 vs. OGD/R 6/12 h).

Based on the results of our *in vivo* experiments, we used HUVECs to construct an OGD/R model *in vitro* to simulate cerebral I/R injury and further explore the effect of metformin in cerebral I/R injury. As shown in Figures 5A,B, HUVECs could upregulate the expression of BDNF under conditions of OGD/R 2, 4, 6, and 8 h/12 h and could significantly upregulate the expression of BDNF under conditions of OGD/R 4, 6, and 8 h/12 h. Selecting OGD/R 6 h/12 h conditions for follow-up studies, we found that metformin could further promote the expression and release of BDNF in HUVECs at a concentration of 20 μM. As the treatment concentration increased, the effect of metformin continued to weaken (Figures 5C–E). Furthermore, as shown in Figures 5F–J, metformin at a concentration of 20 μM promoted the proliferation of HUVECs and reduced cell apoptosis under OGD/R conditions.

### Metformin Promoted HUVECs to Express BDNF Under OGD/R Conditions *via* the AMPK/CREB Pathway *in vitro*

**FIGURE 6:**
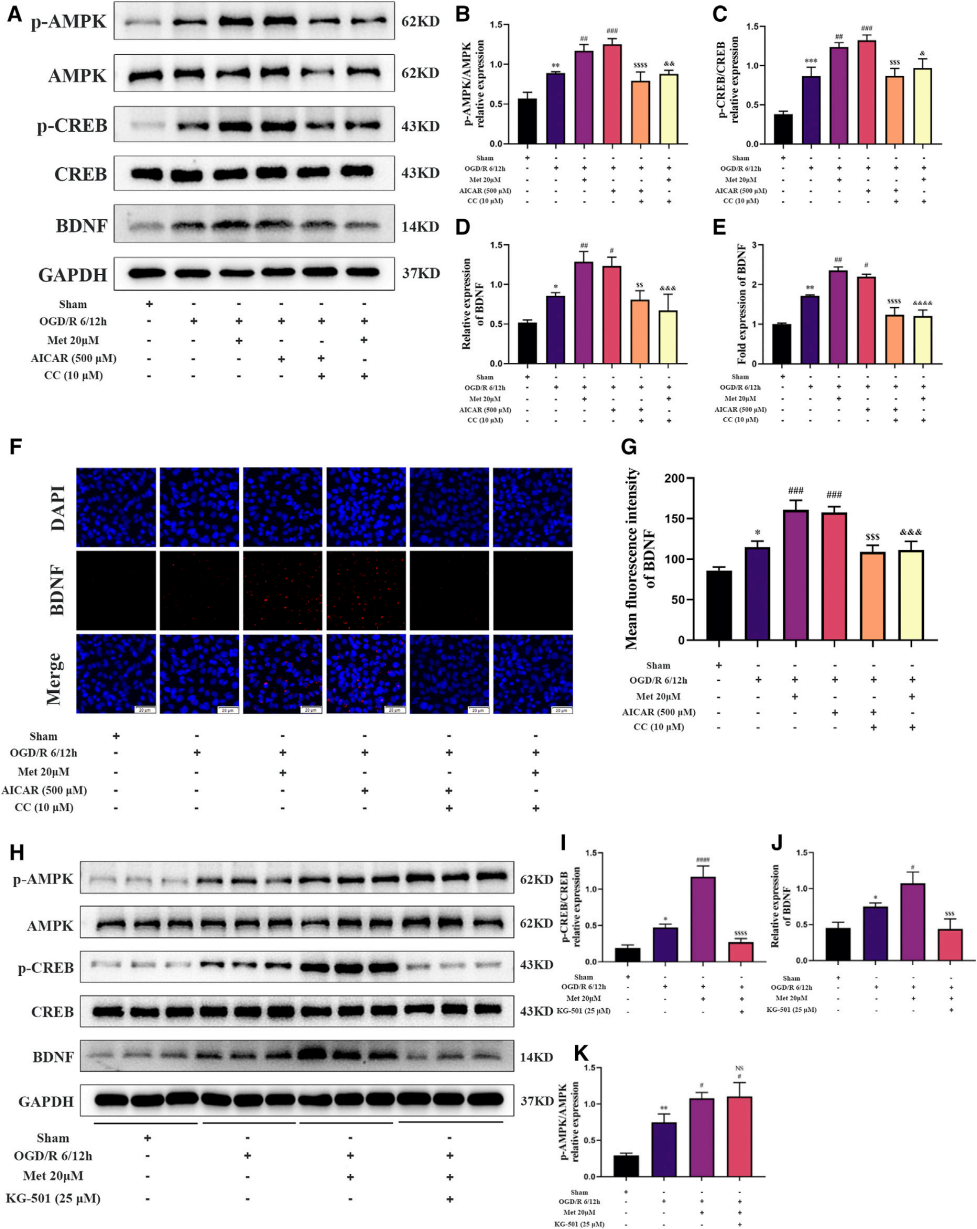
Metformin promotes the expression of BDNF in HUVECs under OGD/R conditions through the AMPK/CREB pathway. **(A–D)** The BDNF expression level and AMPK and CREB phosphorylation levels in HUVECs treated with metformin, AICAR, and Compound C under OGD/R conditions detected were by western blotting (*n* = 3). **(E)** The BDNF transcription level under the above conditions was detected by RT-qPCR (n = 4). **(F,G)** Immunofluorescence staining and quantification of BDNF under the above conditions (*n* = 3, bar = 20 μM). **(H–K)** BDNF expression level and AMPK and CREB phosphorylation levels in HUVECs treated with metformin and KG-501 under OGD/R conditions were detected by western blotting (*n* = 3). (^*^ *p* < 0.05, ^**^ *p* < 0.01, ^***^ *p* < 0.001 vs. sham; ^#^ *p* < 0.05, ^##^ *p* < 0.01, ^###^ *p* < 0.001, ^####^ *p* < 0.0001 vs. OGD/R 6/12 h; NS, not significant, ^$$^ *p* < 0.01, ^$$$^ *p* < 0.001, ^$$$$^ *p* < 0.0001 vs. OGD/R 6/12 h + AICAR; ^&^ *p* < 0.05, ^&&^ *p* < 0.01, ^&&&^ *p* < 0.001, ^&&&&^ *p* < 0.0001 vs. OGD/R 6/12 h + Met 20 μM).

We then explored whether AMPK and CREB were involved in the regulation of BDNF expression in HUVECs by metformin under OGD/R conditions. We treated HUVECs with metformin, AICAR (an AMPK activator), Compound C, and KG-501 (a CREB inhibitor) at the beginning of the reoxygenation period. Western blotting revealed that metformin and AICAR significantly increased the expression of BDNF and phosphorylation levels of AMPK and CREB at the same time, while Compound C inhibited the positive effect of metformin and AICAR (Figures 6A–D). Immunofluorescence staining of the cell slides also revealed that metformin and AICAR could further upregulate BDNF expression in HUVECs, while Compound C reversed the effect of metformin and AICAR (Figures 6F,G). Moreover, quantitative PCR results showed that metformin and AICAR promoted BDNF expression at the transcription level, and Compound C exerted an inhibitory effect also at the transcription level (Figure 6E). We also found that KG-501 could inhibit the increase in BDNF expression and CREB phosphorylation caused by metformin but had no effect on the AMPK phosphorylation level increased by metformin (Figures 6H–K). This suggests that AMPK is located upstream of CERB. Therefore, we concluded that metformin regulated phosphorylation of CREB and further regulated BDNF expression by affecting the phosphorylation level of AMPK in HUVECs under OGD/R conditions. In brief, metformin promotes BDNF expression in HUVECs under OGD/R conditions through the AMPK/CREB pathway.

### BDNF Promoted the Proliferation of SH-SY5Y Cells and Inhibited Cell Apoptosis Under OGD/R Conditions *in vitro*

**FIGURE 7:**
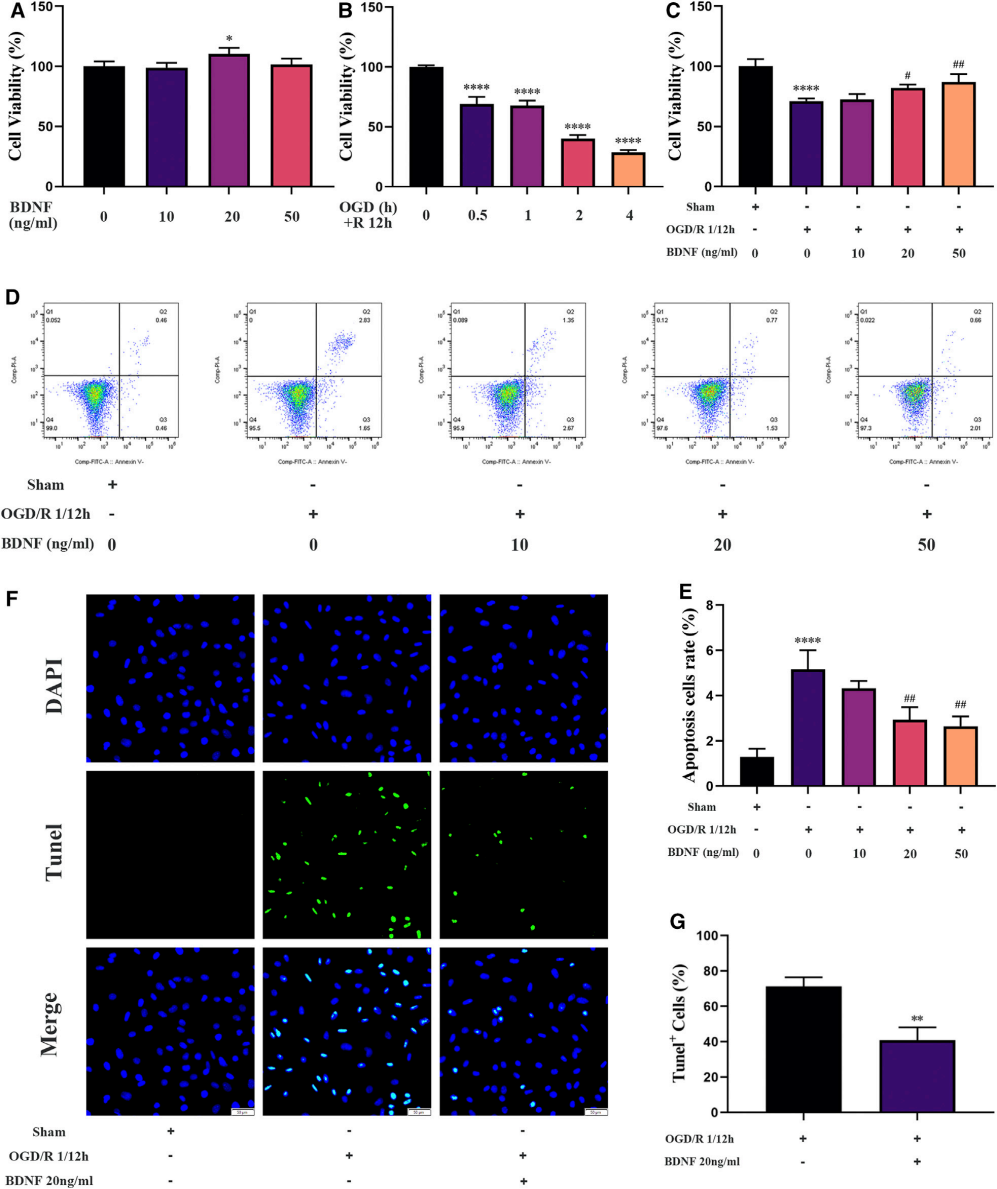
BDNF promotes the proliferation of SH-SY5Y cells and inhibits cell apoptosis under OGD/R conditions. **(A–C)** The effect of BDNF on the proliferation of SH-SY5Y cells was detected by CCK-8 assay under OGD/R conditions (*n* = 4). **(D–G)** The effect of BDNF on apoptosis of SH-SY5Y cells was detected by TUNEL staining (*n* = 3, bar = 50 μm) and flow cytometry (*n* = 3). (^*^ *p* < 0.05, ^**^ *p* < 0.01, ^****^ *p* < 0.0001 vs. sham; ^#^ *p* < 0.05, ^##^ *p* < 0.01 vs. OGD/R 1/12 h).

To explore the protective mechanism of BDNF on neurons under OGD/R conditions, we constructed an *in vitro* OGD/R model using SH-SY5Y cells and treatment with BDNF at the beginning of the reoxygenation period. After the intervention, the proliferation of SH-SY5Y cells was detected using the CCK-8 assay. The results revealed that the conditions of OGD/R 0.5, 1, 2, and 4 h/12 h dramatically reduced the proliferation of SH-SY5Y cells, and OGD/R 1 h/12 h treatment was selected for follow-up studies (Figure 7B). After treatment with various concentrations of BDNF, the CCK-8 assay revealed that the proliferation of SH-SY5Y cells was dramatically increased under OGD/R conditions when the BDNF concentration was 20 or 50 ng/ml (Figure 7C). Apoptosis of SH-SY5Y cells was detected using TUNEL staining and flow cytometry. The flow cytometry results revealed that BDNF concentrations of 20 and 50 ng/ml significantly reduced OGD/R-induced apoptosis in SH-SY5Y cells (Figures 7D,E). Furthermore, TUNEL staining revealed that when the concentration of BDNF was 20 ng/ml, apoptosis of SH-SY5Y cells under OGD/R 1 h/12 h conditions was significantly reduced (Figures 7F,G). Therefore, BDNF could promote the proliferation of SH-SY5Y cells and reduce apoptosis under OGD/R conditions.

### HUVECs Treated With Metformin Alleviated Apoptosis of SH-SY5Y Cells Under OGD/R Conditions *in vitro*

**FIGURE 8:**
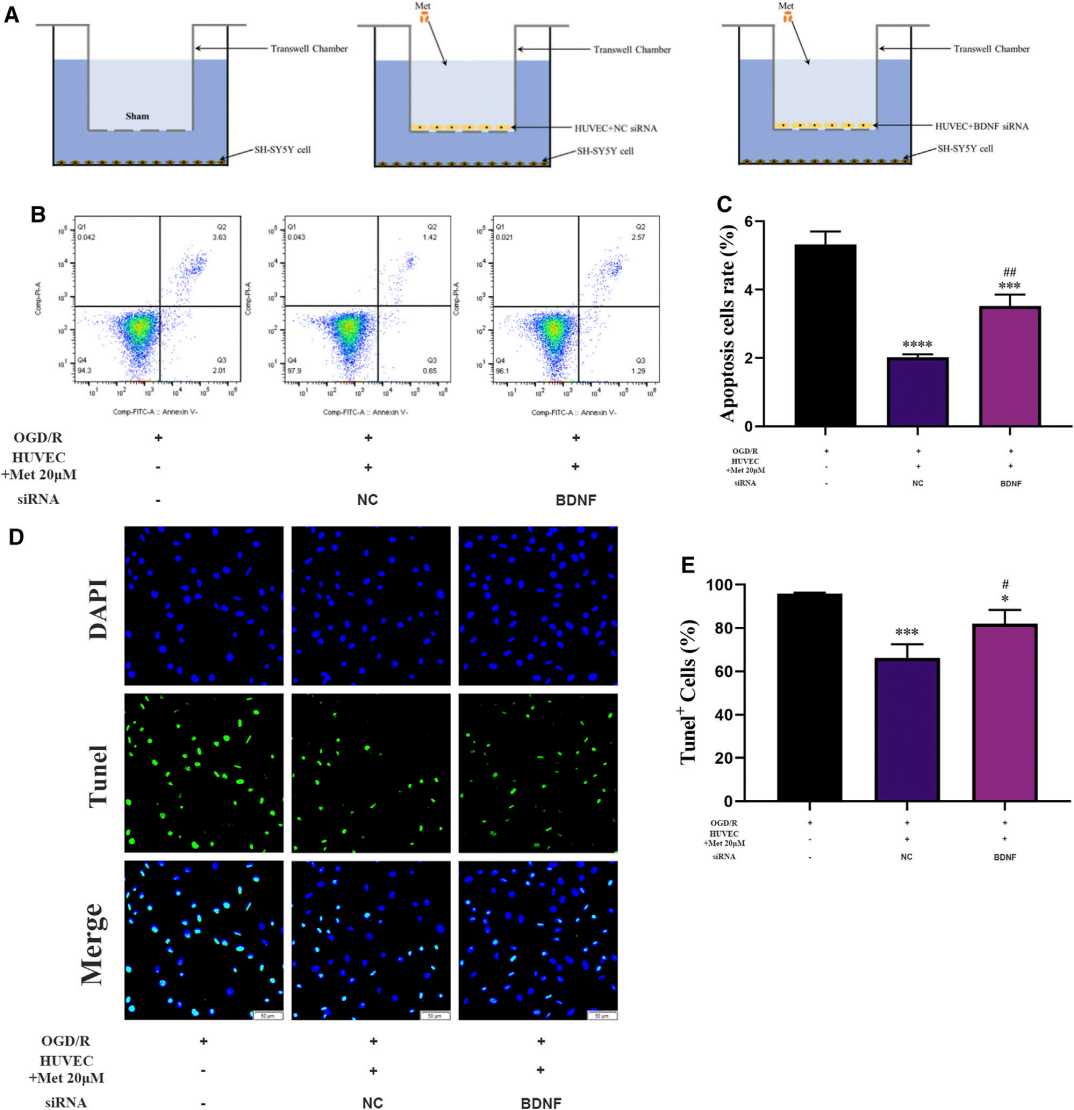
HUVECs treated with metformin reduce apoptosis of SH-SY5Y cells under OGD/R conditions. **(A)** Schematic diagram of grouping in the Transwell chamber assay. **(B–E)** Apoptosis of SH-SY5Y cells was detected by TUNEL staining (*n* = 3, bar = 50 μm) and flow cytometry (*n* = 3). (^*^ *p* < 0.05, ^***^ *p* < 0.001, ^****^ *p* < 0.0001 vs. OGD/R; ^#^ *p* < 0.05, ^##^ *p* < 0.01 vs. OGD/R + HUVEC + Met 20 μM + NC siRNA; NC, negative control).

We examined the function of metformin-treated HUVECs on apoptosis of SH-SY5Y cells using a Transwell chamber assay under OGD/R conditions. Flow cytometry revealed that apoptosis of SH-SY5Y cells could be alleviated in the metformin-treated HUVEC group (Figures 8B,C). Consistent with the above results, TUNEL staining showed that metformin-treated HUVECs could reduce the number of SH-SY5Y cells positive for TUNEL staining more effectively than in the control group (Figures 8D,E). However, the improved apoptosis level of SH-SY5Y cells *via* metformin-treated HUVECs was partially reversed by transfecting BDNF siRNA in HUVECs. Therefore, metformin may decrease the apoptosis of SH-SY5Y cells partially by upregulating BDNF expression in HUVECs under OGD/R conditions.

## Discussion

**FIGURE 9:**
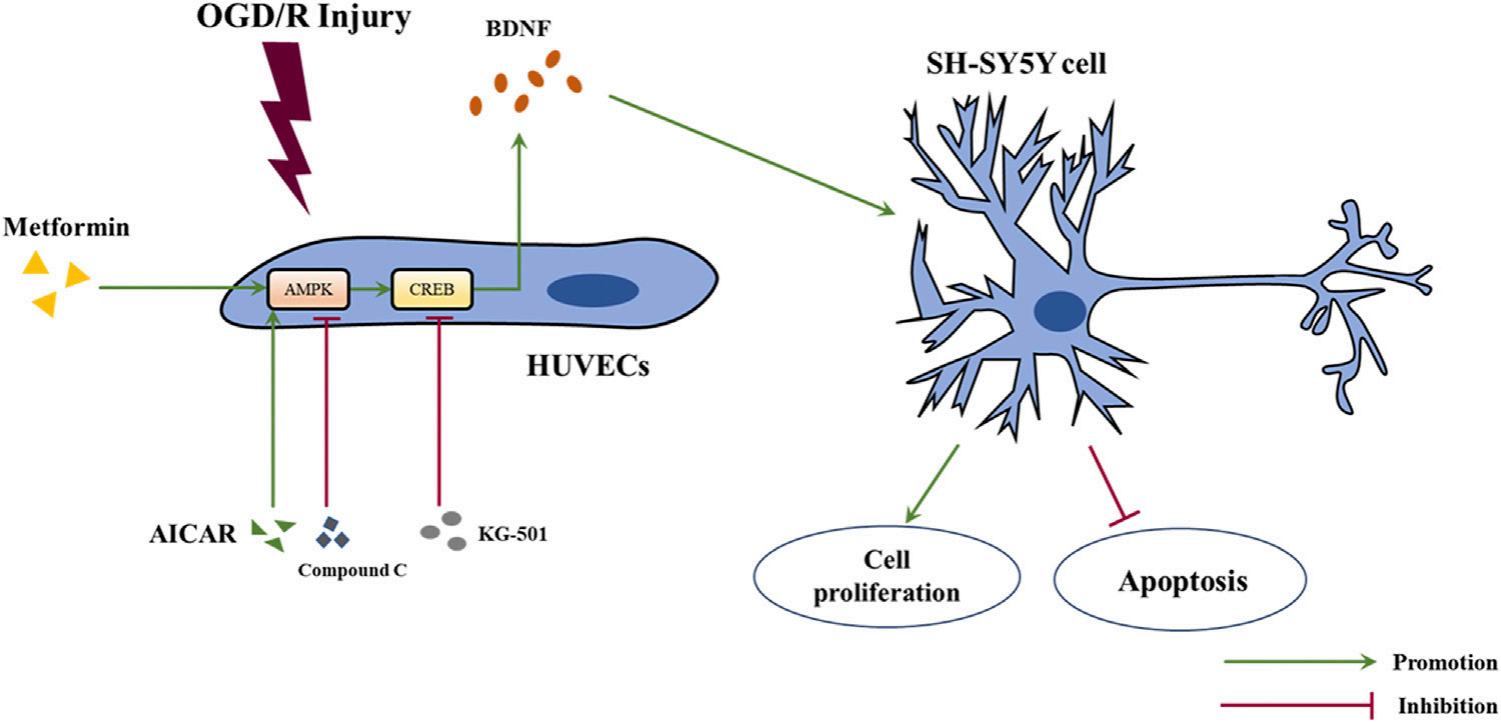
Schematic diagram of the mechanism of metformin in this study. Abbreviations: OGD/R, oxygen-glucose deprivation/reoxygenation; HUVECs, human umbilical vein endothelial cells; BDNF, brain-derived neurotrophic factor.

In this study, we revealed that acute treatment with low-dose metformin upregulated the expression of BDNF in ECs in the ischemic penumbra area in an AMPK-dependent manner, thereby improving cerebral edema, cerebral infarct volume, the neurological deficit score, and neuronal apoptosis caused by cerebral I/R injury *in vivo*. Metformin also promoted BDNF expression in HUVECs *via* the AMPK/CREB pathway, thereby promoting the proliferation of SH-SY5Y cells and reducing cell apoptosis under OGD/R conditions *in vitro*. The mechanism of metformin observed in this research is summarized in Figure 9.

AMPK is the main energy homeostasis regulator in the body and can be activated by phosphorylation when the AMP/ATP ratio increases (Hardie et al., 2012). Many studies have suggested that AMPK is an important mediator in the pathogenesis of stroke (Manwani and Mccullough, 2013). Similarly, we found that AMPK could be activated by phosphorylation both in cerebral I/R injury *in vivo* and under OGD/R conditions *in vitro* and that treatment with metformin can further promote phosphorylation of AMPK. Moreover, we found that the active influence of metformin in cerebral I/R injury and promotion of BDNF expression in HUVECs under OGD/R conditions depend on the activation of AMPK, because the above-mentioned effects of metformin are all inhibited by Compound C.

As a transcription factor, CREB is widely expressed in various organs and participates in the proliferation, differentiation, and survival of various cell types (Kitagawa, 2007). Related studies have reported that CREB could be activated by phosphorylation in the ischemic penumbra area in a model of focal cerebral ischemia (Tanaka et al., 1999). Moreover, many studies have suggested that the activation of CREB phosphorylation can have a neuroprotective effect in models of cerebral ischemia (Miyata et al., 2001; Lee et al., 2004), which is consistent with our results. Furthermore, our *in vitro* data showed that the activation of CREB depends on the activation of AMPK phosphorylation; that is, AMPK is located upstream of CREB. Because AMPK agonists and inhibitors can upregulate and downregulate the phosphorylation level of CREB, but CERB inhibitors cannot affect the phosphorylation level of AMPK (Figure 6).

BDNF, as a neurotrophic factor, is widely distributed throughout the brain, especially in the hippocampus and cortex, and has a wide range of neuroprotective and neurotrophic functions (Eyileten et al., 2021). More and more studies have revealed that BDNF can upregulate and exert neuroprotective effects in cerebral ischemia (Ferrer et al., 2001; Zhang and Pardridge, 2001). A study by Dmitrieva et al. found that in acute ischemic stroke, the body’s endogenous self-protection mechanism can be stimulated to increase the expression of endogenous BDNF, thereby promoting repair of damaged neurons; however, this compensation cannot completely offset the neuronal damage caused by ischemia (Dmitrieva et al., 2010). Similarly, our study found an increased expression of BDNF in both the cortex of the ischemic penumbra in cerebral I/R injury *in vivo* and in HUVECs under OGD/R conditions *in vitro* and that metformin could further promote the expression of BDNF. Increased BDNF expression in the cortex of the ischemic penumbra was accompanied by the improvement in cerebral I/R injury *in vivo*, which suggests that metformin reduces cerebral I/R injury by promoting BDNF expression.

As a first-line hypoglycemic agent, metformin not only has a good hypoglycemic effect (Sanchez-Rangel and Inzucchi, 2017) but can also reduce the risk of cardiovascular (Ghotbi et al., 2013) and cerebrovascular (UK Prospective Diabetes Study (UKPDS) Group, 1998) events and improve the prognosis of stroke patients (Mima et al., 2016). Previous studies have suggested that metformin could play a positive role in neurodegenerative conditions, such as Parkinson’s disease (Fitzgerald et al., 2017; Mor et al., 2020) and Alzheimer’s disease (Luchsinger et al., 2016; Farr et al., 2019; Xu et al., 2021). In the present study, we found that acute treatment with low-dose metformin (10 mg/kg) reduced cerebral I/R injury in a rat model of tMCAO. Interestingly, acute treatment with a low concentration of metformin (20 μM) promoted BDNF expression in HUVECs under OGD/R conditions *in vitro*, while BDNF expression continued to decrease as the concentration of metformin increased. This implies that acute treatment with metformin can only exert a protective effect at low concentrations.

Moreover, metformin not only reduces cerebral I/R injury in a global cerebral ischemia model (Ashabi et al., 2014; Ashabi et al., 2015; Ge et al., 2017) but also has a neuroprotective effect in a model of focal cerebral ischemia (Li et al., 2010; Venna et al., 2014). The above findings were also observed under long-term chronic metformin treatment. A study by Li et al. revealed that acute treatment with metformin aggravated brain damage in the focal ischemia model, which seems to be inconsistent with our experimental results (Li et al., 2010). There are several possible explanations for these conflicting findings. First, the dose of metformin used in our experiment was 10 mg/kg, which is much smaller than the 100 mg/kg dose used in the study by Li et al. Treatment with the various concentrations of metformin resulted in differences in the degree of AMPK activation. Moderate AMPK activation can counteract cerebral ischemic damage by strengthening catabolic pathways and reducing ATP consumption (Manwani and Mccullough, 2013; Jiang et al., 2018). A study by Jiang et al. found that pretreatment with acute low-dose metformin can moderately activate AMPK and has a neuroprotective effect in a model of focal cerebral ischemia (Jiang et al., 2014). Moreover, high-dose metformin has been shown to markedly enhance AMPK activation and lactic acid accumulation, promote ATP consumption, and ultimately aggravate neuronal death (Jiang et al., 2018). Second, metformin was administered at the beginning of the reperfusion period in our study, while it was administered as preconditioning before ischemia in the study by Li et al. Our treatment was a therapeutic intervention, whereas theirs was a preventive intervention, and different intervention times sometimes have different results.

Interestingly, BDNF siRNA transfection in HUVECs was able to partially reverse the metformin-dependent increase in SH-SY5Y cells apoptosis under OGD/R conditions based on the results of the Transwell chamber assay. This result indicates that metformin can reduce the apoptosis level of SH-SY5Y cells by promoting the BDNF expression of HUVECs but also through other currently unclear mechanisms. In addition, according to the ELISA results of the metformin-treated HUVECs culture supernatant, BDNF concentration was approximately 66 pg/ml, while our *in vitro* experiments showed that BDNF could reduce apoptosis levels of SH-SY5Y cells at 20 ng/ml under OGD/R conditions. These results also indicate that metformin not only promoted the BDNF expression of HUVECs, but also enhanced the protective effect of BDNF through an uncertain mechanism. This will be further explored in our future studies.

This research also has many shortcomings. First, we only focused on the effects of metformin in the acute phase and did not investigate the effects and mechanisms of chronic low-dose metformin. We will address these issues in future experiments. Second, we only explored whether BDNF promoted the proliferation of SH-SY5Y cells and reduced apoptosis under OGD/R conditions but not the specific mechanism of BDNF functions, which will be also one of our later research focuses. Finally, the intraperitoneal injection method used in in vivo experiments can greatly reduce the first-pass effect of the drug, but it is also more invasive. Therefore, the protective effects of oral metformin against ischemic stroke should be investigated.

In conclusion, this study shows that acute treatment with low-dose metformin can upregulate BDNF expression in the ischemic penumbra *via* the AMPK/CREB pathway, thereby reducing cerebral I/R injury. The ability of exogenous BDNF to enter the brain tissue is extremely limited because of the blood-brain barrier; this study may lead to a new and effective way of activating endogenous BDNF. Furthermore, our findings provide new evidence and support for metformin as a treatment that can be synchronized with the revascularization of acute cerebral infarction.

## Data Availability

The original contributions presented in the study are included in the article/Supplementary Material, further inquiries can be directed to the corresponding authors.
