# Corrigendum: Acute Administration of Metformin Protects Against Neuronal Apoptosis Induced by Cerebral Ischemia-Reperfusion Injury *via* Regulation of the AMPK/CREB/BDNF Pathway

**DOI:** 10.3389/fphar.2022.929835

**Published:** 2022-06-02

**Authors:** Ke Liu, Lulu Li, Zhijun Liu, Gang Li, Yanqing Wu, Xingjun Jiang, Mengdie Wang, Yanmin Chang, Tingting Jiang, Jianheng Luo, Jiahui Zhu, Hongge Li, Yong Wang

**Affiliations:** ^1^ Department of Neurology, Union Hospital, Tongji Medical College, Huazhong University of Science and Technology, Wuhan, China; ^2^ Department of Neurology, People’s Hospital of Zhengzhou, People’s Hospital of Henan University of Chinese Medicine, Zhengzhou, China

**Keywords:** cerebral ischemia-reperfusion injury, metformin, AMP-activated protein kinase, neuronal apoptosis, brain-derived neurotrophic factor

In the published article, there was an error in **Affiliation 1**. Instead of “Department of Neurology, Tongji Medical College, Union Hospital, Huazhong University of Science and Technology, Wuhan, China,” it should be “Department of Neurology, Union Hospital, Tongji Medical College, Huazhong University of Science and Technology, Wuhan, China.”

The authors apologize for this error and state that this does not change the scientific conclusions of the article in any way. The original article has been updated.

