# Supplementary figures and images for: Acute Administration of Metformin Protects Against Neuronal Apoptosis Induced by Cerebral Ischemia-Reperfusion Injury via Regulation of the AMPK/CREB/BDNF Pathway
Source: Front Pharmacol. 2022 Apr 1;13:832611. doi: 10.3389/fphar.2022.832611 (PMC9010658; doi:10.3389/fphar.2022.832611)

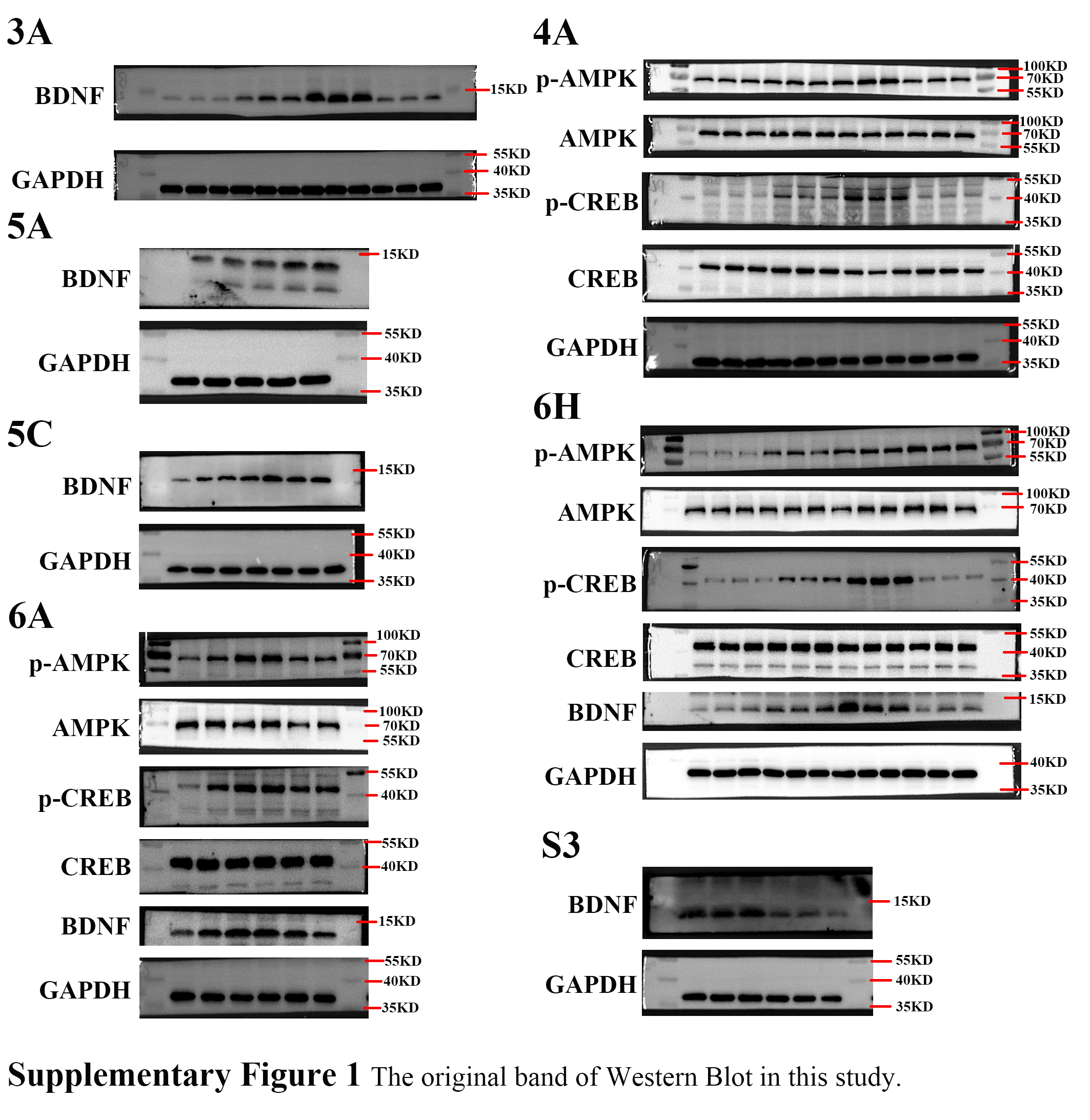

Supplement: Supplementary file 1 [file DataSheet1.ZIP › Supplementary Figure 1.tif]

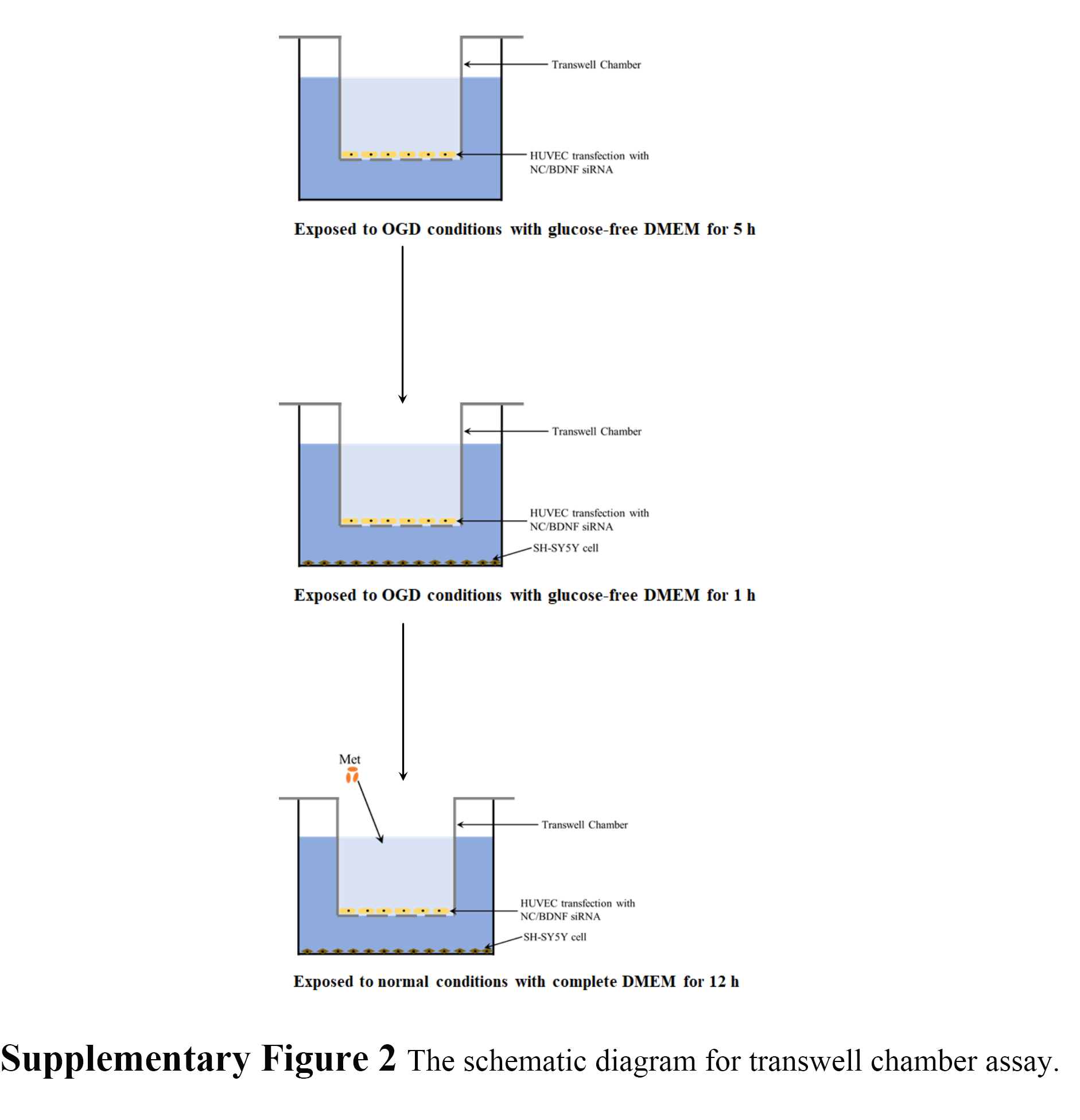

Supplement: Supplementary file 1 [file DataSheet1.ZIP › Supplementary Figure 2.tif]

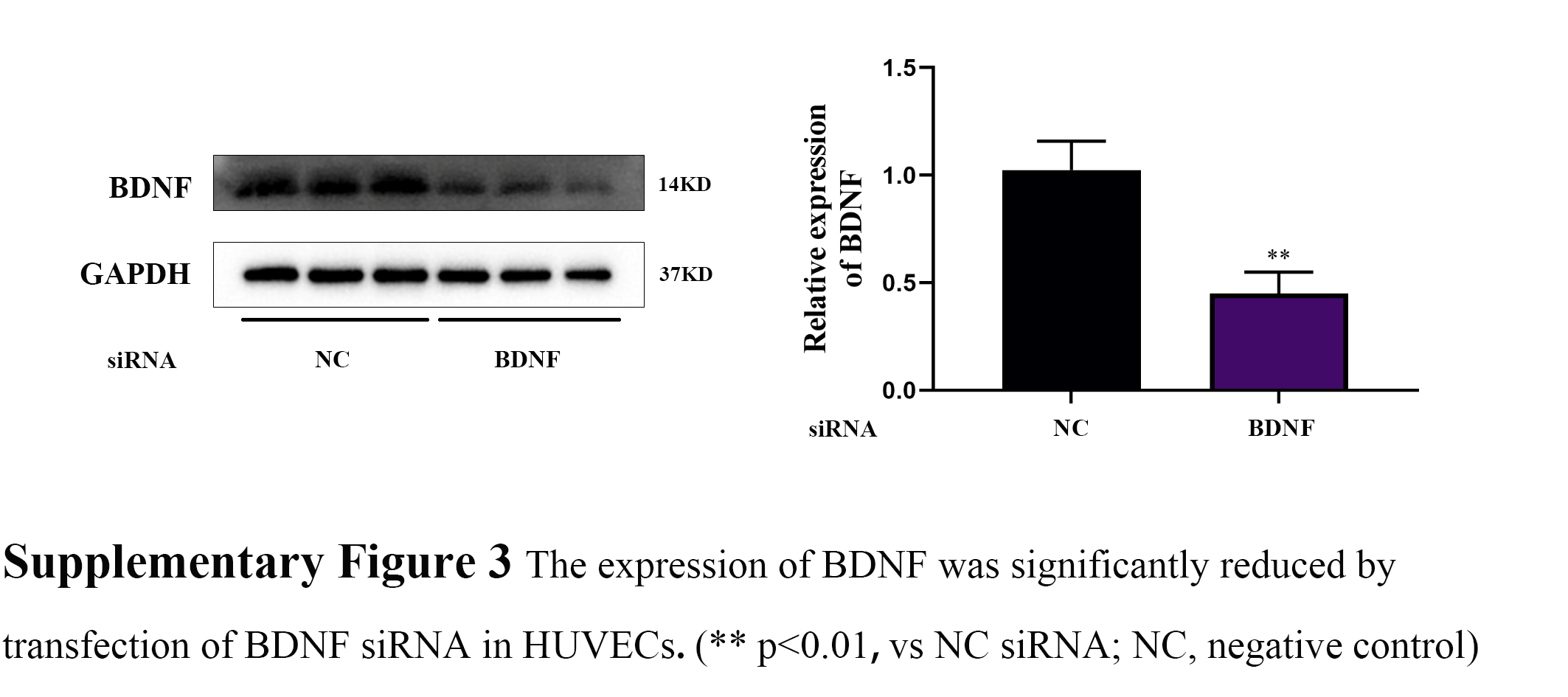

Supplement: Supplementary file 1 [file DataSheet1.ZIP › Supplementary Figure 3.tif]
